# Horizontal Platelet-Rich Fibrin Membrane Block for Peri-Implant Soft Tissue Augmentation: An Experimental Animal Study with Clinical Illustration

**DOI:** 10.3390/dj14030141

**Published:** 2026-03-04

**Authors:** Hao Zeng, Yan Wei, Shimin Yu, Xiaoxin Zhang, Yun Qiu, Richard J. Miron, Yulan Wang, Yufeng Zhang

**Affiliations:** 1 State Key Laboratory of Oral & Maxillofacial Reconstruction and Regeneration, Key Laboratory of Oral Biomedicine Ministry of Education, Hubei Key Laboratory of Stomatology, School & Hospital of Stomatology, Wuhan University, Wuhan 430079, China; hao.zeng@whu.edu.cn (H.Z.);; 2 Department of Oral Implantology, School & Hospital of Stomatology, Wuhan University, 237 Luoyu Road, Wuhan 430079, China; 3 Department of Periodontology, University of Bern, CH-3010 Bern, Switzerland; 4 Oral Biomaterials and Application Technology Engineering Research Center of Hubei Province, Wuhan 430079, China

**Keywords:** horizontal centrifugation, platelet-rich fibrin, peri-implant, soft tissue augmentation

## Abstract

**Background/Objectives:** Adequate peri-implant soft tissue thickness is essential for long-term peri-implant health and esthetics. Horizontal platelet-rich fibrin (H-PRF) has been proposed to support soft tissue regeneration; however, experimental and translational evidence for its application in peri-implant soft tissue augmentation remains limited. This study aimed to evaluate a H-PRF membrane block approach primarily through an experimental animal model, with clinical cases presented to illustrate translational feasibility. **Methods:** A customized compression device was used to fabricate the H-PRF membrane block. The biological performance of the H-PRF membrane block was first evaluated in a rabbit model, with histologic assessment of peri-implant soft tissue thickness and integration at 8 weeks. Representative clinical cases requiring peri-implant mucosal thickening were subsequently treated with H-PRF membrane block on the buccal aspect of the alveolar bone beneath a supra-periosteal flap to demonstrate clinical applicability. **Results:** In the animal model, the H-PRF membrane block resulted in a significant increase in peri-implant soft tissue thickness by increasing the lamina propria compared with control sites demonstrated by histologic analysis. The clinical illustrations showed stable buccal soft tissue volume and contour with minimal patient morbidity. **Conclusions:** Within the limitations of this experimental study, the horizontal H-PRF membrane block technique demonstrated promising biological performance for peri-implant soft tissue augmentation in an animal model. The accompanying clinical illustrations support the translational feasibility of this approach. Clinical relevance: This experimental study provides biological and translational insight into a minimally invasive strategy for peri-implant soft tissue thickening and may inform future controlled clinical investigations.

## 1. Introduction

Soft tissue management has become an essential aspect of modern implant dentistry, directly influencing peri-implant health, long-term tissue stability, and esthetic outcomes [1]. Inadequate peri-implant mucosal thickness is associated with increased risk of marginal bone loss, soft tissue recession, and compromised esthetics [2]. Connective tissue grafting (CTG) has long been recognized as the gold standard for peri-implant soft tissue thickening due to the predictable clinical outcome [3,4]. However, this procedure is associated with several drawbacks, including donor site morbidity, increased surgical time, and patient discomfort. In an effort to overcome these limitations, various alternative materials have been introduced [3]. Although allogeneic materials, such as acellular dermal matrices, and xenogeneic collagen-based matrices eliminate donor site morbidity, their relatively high cost and the uncertainty regarding long-term volumetric stability remain important concerns [3]. Therefore, there is a continued interest in developing minimally invasive, biologically compatible, and patient-friendly techniques to achieve predictable peri-implant soft tissue thickening.

Platelet-rich fibrin (PRF) is an autologous a biomaterial that has gained much attention for soft tissue regeneration due to its inherent fibrin scaffold, growth factors, biocompatibility, and ability to promote wound healing [5,6]. Traditional PRF protocols typically employ fixed-angle centrifugation, which can result in heterogeneous fibrin architecture and inconsistent cellular content. More recently, horizontal centrifugation PRF (H-PRF) has been developed to optimize the separation of blood components and increase the concentration of platelets, leukocytes, and growth factors within the scaffold [7,8,9,10,11]. This approach yields an H-PRF clot or liquid H-PRF with improved biological properties, enhanced cellular viability, and potentially superior regenerative capacity, showing promise in soft tissue regeneration [12]. Despite these advances, the lack of standardization in PRF preparation and application has resulted in inconsistent clinical outcomes, and its direct use for peri-implant soft tissue thickening still lacks standardized protocols and robust clinical evidence [6]. To address this gap, we proposed a unified and reproducible H-PRF membrane block technique, and evaluated its effectiveness for peri-implant soft tissue thickening in both experimental and clinical settings.

This clinical innovation report presents a novel H-PRF membrane block technique in enhancing peri-implant mucosal thickness with minimal invasiveness. The efficacy of this approach was first validated in a rabbit model through histologic analysis, and subsequently translated to human clinical cases with visual evaluation. By combining preclinical and clinical evidence, this report aims to provide a comprehensive overview of the potential benefits and clinical applicability of H-PRF membrane block in peri-implant soft tissue augmentation.

## 2. Materials and Methods

### 2.1. Study Design

This study consisted of two sequential parts. The first part was a preclinical experimental study using a rabbit palatal soft tissue thickening model, designed to evaluate tissue responses and to obtain histological evidence of soft tissue augmentation. The second part was a confirmatory clinical case series, in which representative clinical cases were presented to validate the translational relevance and clinical feasibility of the experimental findings.

### 2.2. The Preparation of the Novel H-PRF Membrane Block

Venous blood was collected from each patient using centrifugation tubes (8–10 mL per tube). Four tubes for solid H-PRF and two tubes for liquid H-PRF (Plasmatrident, Hubei Plasmatrident Biotechnology Co., Ltd., Wuhan, China) were prepared. The tubes were immediately processed using a horizontal centrifuge machine (Plasmatrident, Hubei Plasmatrident Biotechnology Co., Ltd., Wuhan, China) at 700× *g* for 8 min [7]. After centrifugation, the upper yellow fibrin clot (solid H-PRF) was carefully separated from the underlying red blood cell layer using blunt dissection with sterile tweezers. The liquid H-PRF was aspirated with a sterile syringe.

**Figure 1:**
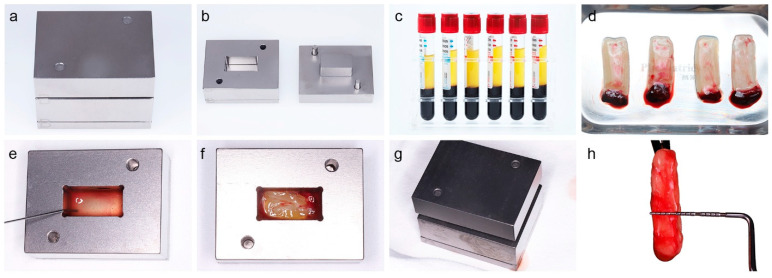
Preparation process of the H-PRF membrane block using a customized mold. (**a**) Overall appearance of the customized membrane block mold; (**b**) Disassembled view of the mold; (**c**) Photograph of the blood sample after centrifugation; (**d**) Illustration showing four H-PRF clots; (**e**,**f**) Illustration showing the process of putting clots into the mold and adding liquid H-PRF in between them; (**g**) Illustration showing the mold being closed for compression; (**h**) Illustration showing the completed H-PRF membrane block.

The obtained H-PRF clots were then transferred into a customized mold (Plasmatrix prelum, Hubei Plasmatrident Biotechnology Co., Ltd., Wuhan, China) (Figure 1) to fabricate H-PRF membrane block. A small amount of liquid H-PRF was added between each layer of the solid H-PRF clots to promote interlayer adhesion. Then the mold was covered with its lid and kept for 2 min to allow polymerization and consolidation. The resulting H-PRF membrane block was subsequently used as a biological soft-tissue thickening material around dental implants.

### 2.3. Animal Experiment

The animal study was conducted prior to the clinical research to validate the effectiveness of the H-PRF membrane block in increasing soft tissue thickness. A total of 10 3-month-old male New Zealand White rabbits (body weight approximately 1.5–2.0 kg) were used. The animal protocol was approved by the Ethics Committee of the Hubei Provincial Academy of Preventive Medicine, Center for Safety Evaluation of Food and Drug (Approval No.: 202340225). The animals were randomly assigned to two groups (n = 5 each): a negative control group (no gingival thickening procedure) and an H-PRF group (gingival thickening using the novel H-PRF membrane block).

Anesthesia was induced by intravenous injection of sodium pentobarbital (30 mg/kg) via the marginal ear vein. After reaching a surgical plane of anaesthesia, 10 mL of whole blood in total was collected from the posterior ear vein and transferred into solid-H-PRF tubes and liquid-H-PRF tubes according to the protocol described above for H-PRF membrane block fabrication.

On the upper maxillary palatal side, a split-thickness flap was elevated, and the H-PRF membrane block was inserted between the palatal flap and the periosteum. The H-PRF membrane block was fixed by two horizontal mattress sutures. Then the flap was closed by interrupted sutures using 5-0 PTFE (polytetrafluoroethylene) sutures. Postoperatively, penicillin was administered for 3 days to prevent infection, and the rabbits were fed a soft diet until sacrifice.

### 2.4. Observation and Evaluation

Postoperative healing of the palatal mucosa was monitored daily during the first week and then every week until sacrifice. The incision sites were inspected for signs of inflammation, wound dehiscence, or infection. Food intake and general activity of the rabbits were recorded to assess postoperative recovery. At 8 weeks after surgery, all animals were euthanized with an overdose injection of sodium pentobarbital. The maxillary specimens, including the palatal mucosa and underlying bone, were harvested en bloc and immediately fixed in 4% paraformaldehyde overnight at 4 °C. After fixation, the specimens were decalcified in 10% ethylenediaminetetraacetic acid (EDTA, pH 7.4) for approximately 4 weeks at room temperature, with the solution changed every 3 days to ensure complete decalcification. Upon completion of the decalcification process, the specimens were carefully rinsed under running tap water to remove any residual EDTA. Following this, the specimens underwent a graded dehydration process through an ascending series of ethanol concentrations (from 70% to 100%) to remove water and prevent tissue distortion during embedding. After dehydration, the samples were infiltrated with molten paraffin wax at 60 °C to facilitate proper tissue embedding. Once adequately infiltrated, the specimens were embedded in paraffin blocks and sectioned longitudinally at a thickness of 5 μm using a microtome. The resulting tissue sections were then mounted onto glass slides and were prepared for subsequent histological evaluation.

Sections were stained with hematoxylin and eosin (H&E) and examined under a light microscope. For H&E staining, paraffin sections were first deparaffinized in xylene and rehydrated through a graded series of ethanol followed by rinsing in distilled water, then the sections were immersed in hematoxylin for 5 min to stain cell nuclei, rinsed thoroughly with running tap water, and briefly differentiated in acid alcohol. The slides were then blued in alkaline water and counterstained with eosin for approximately 2 min to visualize the cytoplasm and extracellular components. After staining, all sections were dehydrated through ascending ethanol concentrations, cleared in xylene, and mounted with a permanent mounting medium. Microscopic examination was carried out using a light to evaluate tissue architecture, epithelial continuity, connective tissue thickness. These findings were compared between the control and H-PRF group at the 8-week endpoint. Representative photomicrographs were captured at standardized magnifications.

For histological analysis, the following parameters were measured using ImageJ software (Version 1.54p, National Institutes of Health, Bethesda, MD, USA):

Epithelial thickness—defined as the distance from the outer epithelial surface to the basal lamina.

Lamina propria thickness—measured as the distance from the basal lamina to the interface with the underlying periosteum.

Measurements were performed at randomly selected sites per section, and the mean value was used for analysis. All histological evaluations were performed by a blinded examiner.

### 2.5. Statistical Analysis

All quantitative data were expressed as mean ± standard deviation (SD). The Shapiro–Wilk test was applied to verify the normality of data distribution. Comparisons between control vs. H-PRF groups were performed using the independent-samples t-test for normally distributed data or the Mann–Whitney U-test for non-parametric data. All statistical analyses were conducted using GraphPad Prism version 10 (GraphPad Software, San Diego, CA, USA). A *p*-value of < 0.05 was considered to indicate statistical significance.

## 3. Results

### 3.1. Animal Experiment Results

All rabbits successfully healed without any adverse events following the experimental procedure. Histological examination of the tissues at the 8-week endpoint revealed that the H-PRF membrane block had been completely resorbed, with no residual material observed. The overall epithelial architecture was well preserved, showing continuous epithelial lining without disruption or ulceration in both groups. The epithelial layer in the H-PRF group appeared slightly thicker compared to the control group, characterized by a modest increase in the thickness epithelial cell layers and a more distinct basal layer arrangement. In contrast, the lamina propria beneath the epithelium showed a remarkable increase in thickness in the H-PRF group. The connective tissue exhibited abundant fibroblasts and newly formed small blood vessels, suggesting active remodeling and angiogenesis in the H-PRF group. Importantly, no signs of inflammatory cell infiltration, necrosis, or foreign-body reaction were detected in any specimens, further confirming the excellent tissue compatibility of the H-PRF membrane block. Collectively, these findings demonstrate that the H-PRF membrane block effectively enhanced connective tissue regeneration and integration without eliciting adverse inflammatory responses.

**Figure 2:**
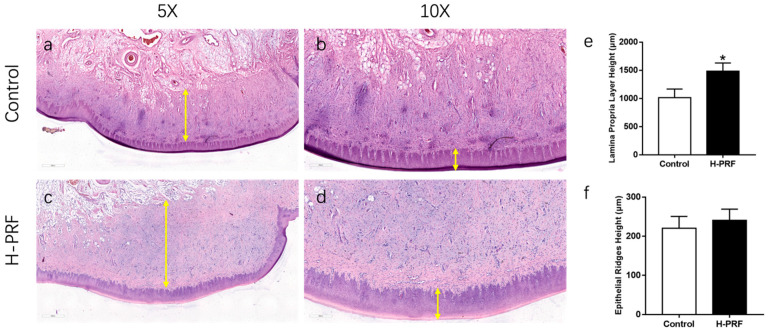
Histological images and quantitative analysis. (**a**) H&E-stained section of the control group at 5× magnification; yellow arrows indicate the lamina propria. (**b**) H&E-stained section of the control group at 10× magnification; yellow arrows indicate the epithelial layer. (**c**) H&E-stained section of the H-PRF group at 5× magnification; yellow arrows indicate the lamina propria. (**d**) H&E-stained section of the H-PRF group at 10× magnification; yellow arrows indicate the epithelial layer. (**e**) Quantitative analysis of lamina propria thickness (bar chart). (**f**) Quantitative analysis of epithelial thickness (bar chart). * denotes (*p* < 0.05).

For the quantitative analysis, the H-PRF membrane block group (240.33 ± 28.84 μm) showed a slight increase compared to the control group (220.33 ± 30.18 μm) (Figure 2), but this difference was not statistically significant (*p* = 0.268). In contrast, the H-PRF membrane block significantly increased the lamina propria thickness, with an average of 1486.50 ± 142.45 μm, compared to 1015.17 ± 151.70 μm in the control group (*p* < 0.05).

### 3.2. Clinical Case Report

Case 1: Peri-implant Soft Tissue Thickness Augmentation of Tooth #36 at Second-Stage Surgery

A 24-year-old male patient presented with a missing tooth #36 and underwent implant placement. After 3 months of healing, the patient returned for second-stage surgery. Prior to the second-stage procedure, a significant buccal soft tissue concavity was observed at the implant site, indicating insufficient peri-implant soft tissue thickness.

**Figure 3:**
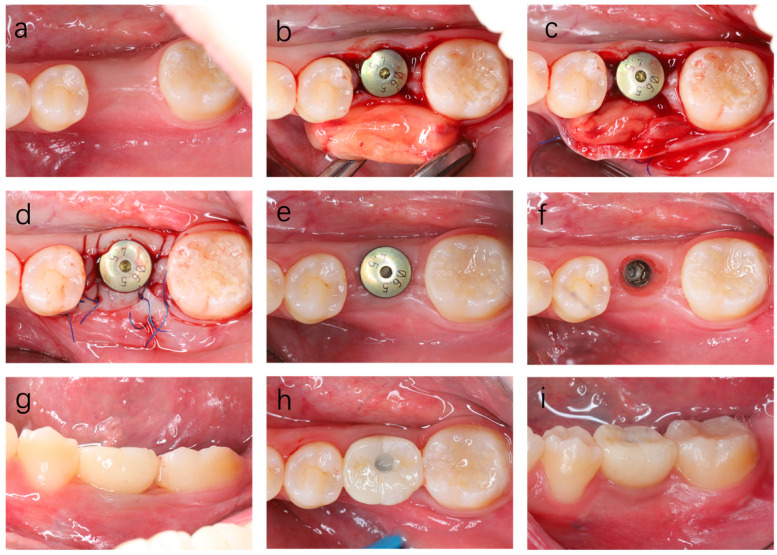
Clinical photographs showing the treatment process of Case 1. (**a**) At the second-stage surgery, a slight buccal soft tissue insufficiency was observed. (**b**) The prepared H-PRF membrane block was placed on the buccal side to assess its size and adaptation. (**c**) The H-PRF membrane block was inserted into the inner side of the buccal supra-periosteal pocket and fixed with sutures. (**d**) The soft tissue was sutured and secured around the healing abutment. (**e**) One month postoperatively, the buccal gingiva appeared well contoured. (**f**) After removing the healing abutment, the peri-implant mucosa showed adequate soft tissue volume. (**g**) Buccal view at crown delivery. (**h**) Occlusal view at crown delivery. (**i**) Buccal view at 1-year follow-up.

The treatment provided to the patient was approved by the Ethics Committee of the School and Hospital of Stomatology, Wuhan University [WDKQ2025(B21)], and was conducted in accordance with the Declaration of Helsinki. Given the poor soft tissue volume, the patient was treated with H-PRF membrane block to augment the buccal soft tissue thickness. A papilla-preserved crestal incision was made from the alveolar crest to replace the healing screw with a healing abutment. A deep buccal supra-periosteal pocket was created via sharp dissection, reaching the gingival mucosal junction at the apex, as well as the mesial and distal teeth (Figure 3).

Venous blood was collected from the patient and processed using the H-PRF protocol described earlier. The resulting H-PRF membrane block was inserted into the pre-established pocket and secured with two sutures at the mesial and distal sites using 5-0 PTFE thread. The buccal flap was then meticulously sutured to the lingual soft tissue around the healing abutment.

Postoperatively, the patient was prescribed antibiotics (amoxicillin + metronidazole; clindamycin in penicillin-allergic patients) and Ibuprofen 600 mg for pain management. Specific postoperative care instructions included cold compresses and rinsing with 0.12% chlorhexidine mouthwash twice daily. Follow-up at 2 weeks showed good healing with no infection or wound dehiscence. At the time of crown delivery, the patient’s buccal tissue contour had been significantly restored. The 1-year follow-up demonstrated stable peri-implant soft-tissue thickening, with well-filled interdental papillae and a harmonious soft-tissue contour.

Case 2: Peri-implant Soft Tissue Thickness Augmentation of Tooth #21, #22 at Second-Stage Surgery

A 27-year-old male patient presented with missing teeth #21 and #22, for which implants were placed during the first stage surgery. After a healing period of 6 months, the patient returned for second-stage surgery. Upon evaluation, it was noted that the patient had insufficient labial soft tissue volume at the implant sites.

**Figure 4:**
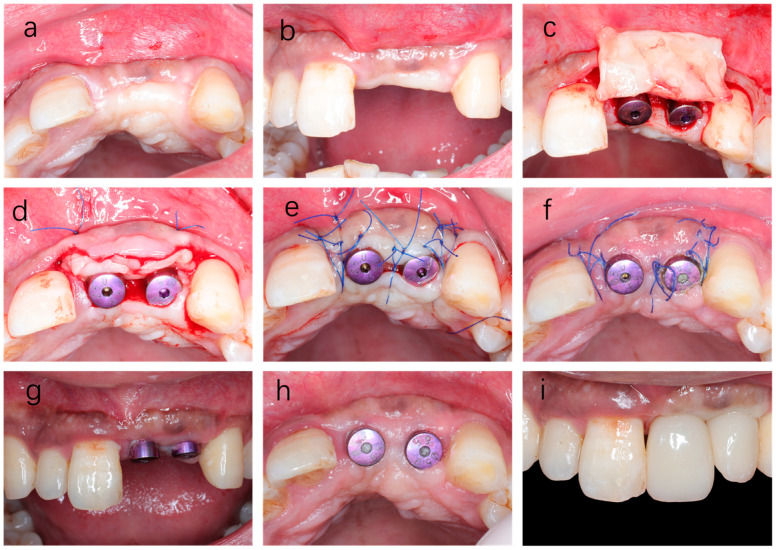
Clinical photographs showing the treatment process of Case 2. (**a**,**b**) At the second-stage surgery, an obvious buccal soft tissue insufficiency was observed. (**c**) The prepared H-PRF membrane block was placed on the labial side to assess its size and adaptation. (**d**) The H-PRF membrane block was inserted into the inner side of the labial supra-periosteal pocket and fixed with sutures. (**e**) The soft tissue was sutured and secured around the healing abutment. (**f**) Two weeks later, the soft tissue healed greatly at the day of suture removal. (**g**,**h**) One month postoperatively, the buccal gingiva appeared well contoured. (**i**) Two months postoperatively, the provisional teeth were delivered.

The patient was treated with H-PRF membrane block to restore the alveolar contour. Similar to Case 1, a papilla-preserved crestal incision was made from the alveolar crest, and the healing screw was replaced with a healing abutment. A deep labial supra-periosteal pocket was carefully created, reaching the gingival mucosal junction at the apex and adjacent mesial and distal teeth (Figure 4).

The H-PRF membrane block was prepared and grafted as mentioned in Case 1, and the patient received the same postoperative care.

## 4. Discussion

The results of the present study demonstrate the effectiveness of the H-PRF membrane block in enhancing peri-implant soft tissue thickness, both in animal experiments and clinical cases. In the animal study, H-PRF significantly increased peri-implant soft tissue thickness, particularly within the lamina propria, compared to the control group. Histological analysis revealed that the H-PRF membrane blocks were fully integrated into the surrounding tissue and completely resorbed by the 8-week endpoint, with no residual material remaining. This suggests that H-PRF has considerable potential in soft tissue regeneration around dental implants. In clinical illustration, the use of the H-PRF membrane block in Case 1 and Case 2 successfully augmented the buccal soft tissue thickness. Follow-up assessments at 1 year showed stable soft tissue volume, with significant restoration of the labial tissue contour and no complications, thus confirming the efficacy and safety of this technique in clinical applications.

The hard palate region of the rabbit was selected for the present study because it features keratinized gingiva, which closely resembles the clinical human gingival phenotype around implants [13], allowing for a more realistic assessment of soft-tissue augmentation techniques. Our study found that H-PRF membrane block primarily increased the thickness of the lamina propria (connective tissue layer) around dental implants, which plays a crucial role in maintaining peri-implant tissue health. The lamina propria provides structural support to the overlying epithelium and facilitates tissue regeneration [14]. Composed of fibroblasts, collagen fibers, and blood vessels, this connective tissue layer is essential for wound healing and tissue stability around implants [15,16]. The fibroblasts in the lamina propria synthesize collagen and other extracellular matrix components, which anchor the mucosa to the underlying bone, promoting tissue stability. Furthermore, the vascularization within the lamina propria is vital for delivering nutrients and oxygen to the peri-implant tissues, crucial for both healing and regeneration [17]. Our research is the first to provide histological evidence demonstrating how platelet concentrates can enhance gingival tissue thickness, particularly by increasing the lamina propria thickness. This finding is particularly significant because it highlights the regenerative potential of H-PRF membrane block as an effective, minimally invasive alternative to traditional soft tissue augmentation techniques, such as connective tissue grafting (CTG).

PRF exerts its regenerative effects through several key mechanisms. Firstly, PRF contains an abundance of platelets and leukocytes, which continuously release a variety of growth factors such as PDGF, VEGF, etc. [5]. These growth factors are known to promote the proliferation, migration, and differentiation of fibroblasts and endothelial cells, accelerating the processes of soft tissue healing and angiogenesis. PRF also forms a three-dimensional fibrin scaffold, which acts as a support structure for cell adhesion, migration, and tissue integration. Moreover, the anti-inflammatory and antimicrobial properties of the leukocytes and cytokines present in H-PRF help reduce postoperative inflammation and create an optimal healing environment [18].

The horizontal centrifugation method used to prepare H-PRF optimizes the separation of blood components, leading to a higher concentration of platelets, leukocytes, and growth factors compared to traditional PRF techniques [9]. This concentration increase enhances the biological activity and regenerative capacity of H-PRF [9], making it an ideal material for peri-implant soft tissue augmentation.

Compared with the gold standard CTG with the drawbacks such as donor site morbidity, increased surgical time, and postoperative discomfort, H-PRF membrane block technique provides a minimally invasive alternative, avoiding the need for second surgery site, reducing surgical time, and facilitating a faster recovery. In addition, H-PRF, being an autologous material, offers a cost-effective solution for soft tissue regeneration compared with biomaterials such as acellular dermal matrices and xenogeneic collagen matrices. Current clinical evidence suggests that PRF can contribute to increased peri-implant mucosal thickness and keratinized mucosa width [19]; however, its efficacy generally remains comparable to or slightly lower than that of the gold-standard autogenous grafts [20,21]. When used in combination protocols—for example, L-PRF together with CTG—some studies have reported short-term improvements in esthetic or biological outcomes compared with SCTG alone [22]. Therefore, PRF may be considered a minimally invasive adjunctive option for soft-tissue enhancement around implants, though it cannot yet replace autogenous tissue grafts. In comparison, our innovation lies in the use of four layers of H-PRF membranes, which are compressed into a single block, providing enhanced stability and long-lasting effects compared to traditional PRF applications. The resulting H-PRF membrane block possesses a certain tensile strength, allowing it to withstand suture traction, thereby demonstrating good operability as a soft-tissue regenerative material. Moreover, as the duration of compression with the mold cover increases, the membrane becomes thinner, enabling clinicians to adjust the compression time according to their specific clinical needs. Based on the authors’ experience, a compression duration of approximately 1–2 min is recommended.

The clinical application of H-PRF membrane blocks demonstrates significant improvements in peri-implant soft tissue contour and mucosal volume, leading to both functional success and aesthetic enhancement for patients. This technique is particularly beneficial for patients with thin biotypes or those requiring implants in the esthetic-zone, where soft tissue volume is crucial for both esthetics and long-term implant success.

Despite promising results, this study has several limitations. Firstly, the sample size in both the animal experiment and clinical cases was small, which may limit the generalizability of the findings. Additionally, the clinical follow-up duration was limited, and longer-term studies (e.g., 2–5 years) are necessary to confirm the long-term stability of soft tissue augmentation and its impact on implant success. Lastly, future studies should compare H-PRF with other established techniques, such as CTG, to better evaluate the relative effectiveness of H-PRF. To further validate the findings, future studies should include larger patient cohorts and multicenter collaborations to confirm the efficacy of the H-PRF membrane block technique.

## 5. Conclusions

• The H-PRF membrane block technique improves the thickness and stability of the lamina propria.
• Compared with traditional soft tissue augmentation methods, it avoids donor site morbidity and reduces surgical time.
• Overall, the H-PRF membrane block technique represents a patient-friendly alternative to connective tissue grafting.

## Data Availability

The original contributions presented in this study are included in the article. Further inquiries can be directed to the corresponding author.
